# Correlates of the support for smoke-free policies among smokers: A cross-sectional study in six European countries of the EUREST-PLUS ITC EUROPE SURVEYS

**DOI:** 10.18332/tid/103918

**Published:** 2019-03-27

**Authors:** Marcela Fu, Yolanda Castellano, Olena Tigova, Ute Mons, Thomas Agar, Christina N. Kyriakos, Anne C. K. Quah, Geoffrey T. Fong, Antigona C. Trofor, Krzysztof Przewoźniak, Witold A. Zatoński, Tibor Demjén, Yannis Tountas, Constantine I. Vardavas, Esteve Fernández, on behalf of the EUREST-PLUS Consortium

**Affiliations:** 1 Catalan Institute of Oncology (ICO), Barcelona, Spain; 2 Bellvitge Biomedical Research Institute (IDIBELL), Barcelona, Spain; 3 University of Barcelona (UB), Barcelona, Spain; 4 Cancer Prevention Unit and WHO Collaborating Centre for Tobacco Control, German Cancer Research Center (DKFZ), Heidelberg, Germany; 5 Department of Psychology and School of Public Health and Health Systems, University of Waterloo (UW), Waterloo, Canada; 6 European Network for Smoking and Tobacco Prevention (ENSP), Brussels, Belgium; 7 University of Crete (UoC), Heraklion, Greece; 8 Ontario Institute for Cancer Research (OICR), Toronto, Canada; 9 University of Medicine and Pharmacy ‘Grigore T. Popa’ Iasi (UMF Iasi), Iasi, Romania; 10 Aer Pur Romania (APR), Bucharest, Romania; 11 Health Promotion Foundation (HPF), Warsaw, Poland; 12 Maria Skłodowska-Curie Institute ‒ Oncology Center (MSCI), Warsaw, Poland; 13 European Observatory of Health Inequalities, President Stanisław Wojciechowski State University of Applied Sciences (PSWZ), Kalisz, Poland; 14 Smoking or Health Hungarian Foundation (SHHF), Budapest, Hungary; 15 National and Kapodistrian University of Athens (UoA), Athens, Greece

**Keywords:** Secondhand exposure, smoke-free policies, smokers, Europe, WHO FCTC

## Abstract

**INTRODUCTION:**

This report describes the support for smoke-free policies in different settings among smokers in six European countries and the relationship between their opinions about the places where smoking should be banned and their beliefs about the harms of secondhand smoke to non-smokers.

**METHODS:**

A cross-sectional survey (the ITC 6 European Country Survey, part of the EUREST-PLUS Project) was conducted using nationally representative samples of adult smokers in Germany, Greece, Hungary, Poland, Romania and Spain (n=6011). We describe the prevalence of agreement and support for smoke-free policies in different settings according to sociodemographics, smoking characteristics and beliefs about the danger of secondhand smoke to non-smokers.

**RESULTS:**

There was high agreement with smoking regulations in cars with preschool children and in schoolyards of primary/secondary schools (>90% overall) and low agreement with banning smoking in outdoor terraces of bars/pubs (8.6%; 95%CI: 7.5%-9.8%) and restaurants (10.1%; 95%CI: 8.9%-11.4%). The highest support for complete smoking bans inside public places came from smokers in Poland, among women, people aged ≥25 years, who had low nicotine dependence, and who tried to quit smoking in the last 12 months. About 78% of participants agreed that tobacco smoke is dangerous to non-smokers, ranging from 63.1% in Hungary to 88.3% in Romania; the highest agreement was noted among women, the 25-54 age groups, those with higher education, low cigarette dependence, and those who tried to quit in the last 12 months. The support for complete smoking bans in public places was consistently higher among smokers who agreed that secondhand smoke is dangerous to non-smokers.

**CONCLUSIONS:**

Smokers in six European countries declared strong support for smoke-free policies in indoor settings and in settings with minors but low support in outdoor settings, particularly leisure facilities. More education is needed to increase the awareness about the potential exposure to secondhand smoke in specific outdoor areas.

## INTRODUCTION

Secondhand smoke is a known risk factor for preventable disease and death worldwide^1^. In 2009, the World Health Organization (WHO) Framework Convention on Tobacco Control (FCTC) promoted the implementation of smoke-free environments^2^ and since then comprehensive legislation in many countries has led to a decrease in smoking in public places, with subsequent reduction in exposure to secondhand smoke^3^.

Knowing the opinions and attitudes of smokers about smoking regulations in different settings is crucial to achieving smoke-free environments. The International Tobacco Control Policy Evaluation Project (the ITC Project; www.itcproject.org/) aims at tracking and comparing the impact of national-level tobacco policies among representative samples of adult smokers in 29 countries. Results have shown increasing support for smoke-free laws among smokers in France^4,5^ and Ireland^6^, and varied support in the UK^7,8^, Germany and the Netherlands^9^.

The ITC Project is a dynamic cohort study worldwide and many countries are continuously joining. In 2014, five new countries plus Germany started up the EUREST-PLUS Project (https://eurestplus.eu), aiming to assess and monitor the impact of the ratification of the WHO FCTC at the European level through the implementation of the European Union (EU) Tobacco Products Directive^10,11^. Following the ITC methodology, a prospective cohort study was established (the ITC 6 European Country Survey or ITC 6E Survey) in Germany, Greece, Hungary, Poland, Romania, and Spain. The implementation of smoke-free legislation in these countries has been heterogeneous^12^. For example, Spain has the more restrictive smoke-free legislation, banning smoking totally in indoor areas of workplaces, enclosed public places, restaurants, bars, healthcare and education facilities, as well as in public transport. Legislation in Hungary allows separate enclosed smoking rooms in healthcare facilities, while in Greece there is a partial smoking ban in bars, having smoking areas or some exceptions in enclosed public places and other workplaces. Poland has a total smoking ban only in enclosed public places and in healthcare facilities, while smoking rooms are allowed in restaurants, bars and other workplaces, airports and waiting halls in bus and train stations. In Romania, a total smoke-free ban exists only in healthcare facilities and in public transportation, while smoking may be allowed in restaurants and bars smaller than 100 m^2^ and enclosed smoking rooms are allowed in larger restaurants and bars, as well as in other workplaces and enclosed public places. Smoke-free legislation in Germany is regulated at the regional level; in most states, separate, enclosed smoking rooms are allowed, while smaller establishments that do not serve food are exempted from the smoking ban altogether. Total smoking bans for restaurants and bars are in place in Saarland, Bavaria and North Rhine-Westphalia^12^.

The objective of this study is to describe the level of support for smoke-free policies among smokers from these six European countries and to examine its relationship with opinions about the harms of secondhand smoke to non-smokers. This work adds new evidence in this regard, with recent and wider information about the support for smoke-free regulations in Europe.

## METHODS

### Design

The EUREST-PLUS ITC 6E Survey includes representative samples of approximately 1000 smokers in Germany, Greece, Hungary, Poland, Romania, and Spain (n=6011). The methods used in the survey are explained elsewhere^11,13^. Briefly, samples of adult current smokers aged 18 years and over (having smoked >100 cigarettes in their lifetime and having smoked at least once in the past 30 days) were recruited, being representative of all geographic regions in each of the six EU Member States (MS). Using a random-walk method, households were randomly selected and were considered to be eligible if they included at least one eligible smoker. Where available, one male and one female smoker were selected from each household using the last birthday method^14^. The current study analysed the data of the inception cohort (Wave 1 data) of the EUREST-PLUS Project that was collected over a month period in each EU MS between June and September 2016. After informed consent was provided, a computer-assisted personal interview (CAPI) was conducted. The study protocol was approved by an ethics committee in each of the participating countries. The participants received a small remuneration for their participation. The sociodemographic characteristics of the sample are provided elsewhere^15^.

### Measures

#### Opinions about smoking in different places

These were determined with the question: ‘At which of the following places do you think smoking SHOULD be allowed?’. The places included were: on outdoor terraces of bars, pubs and restaurants, within 5 m of entrances to public buildings, in cars with pre-school children, in cars with non-smokers, in schoolyards of primary and secondary schools, at beaches, in open stadiums, at bus stops, and in subway and train stations. The possible answers were: ‘strongly support’, ‘support’, ‘oppose’ and ‘strongly oppose’, which were recoded as ‘support’ (first and second possible answers) and ‘oppose’ (third and last possible answers).

#### Support for a complete smoking ban inside public places

This was determined with the question: ‘Do you support or oppose a complete smoking ban in…?’. The places enquired were: hospitals and healthcare facilities, inside restaurants, drinking establishments such as pubs and bars, and entertainment establishments such as nightclubs and discos. The possible answers were: ‘strongly support’, ‘support’, ‘oppose’ and ‘strongly oppose’, which were recoded as ‘support’ (the first two possible answers) and ‘oppose’ (the last two possible answers).

#### Belief about the harmfulness of secondhand smoke to non-smokers

This was determined with the question: ‘Do you strongly agree, agree, neither agree nor disagree, disagree, or strongly disagree with the following statement: “Cigarette smoke is dangerous to nonsmokers”? ’. The answers were categorised as ‘agree’ (those who answered ‘strongly agree’ and ‘agree’), ‘neither agree nor disagree’, and ‘disagree’ (those who answered ‘disagree’ or ‘strongly disagree’).

### Analysis

We computed the prevalence and 95% confidence intervals (CI) of the support for smoking regulations in different public settings (responses ‘oppose’ to allowing smoking in different places) overall and by the six EU MS. We also described the prevalence (and 95% CI) of support for a complete smoking ban in healthcare facilities, restaurants, pubs/bars and entertainment settings overall and by country, sociodemographic (sex, age, educational level) and smoking characteristics (nicotine dependence measured with the Heaviness of Smoking Index16 and quit attempts in the last 12 months). We similarly described the prevalence of the degree of agreement with the belief about the harms of secondhand smoke to non-smokers. Finally, we analysed the relationship between the respondents’ opinions about the places where smoking should not be allowed, as well as their support for complete smoking bans in public places, according to the degree of agreement with the belief about the harms of secondhand smoke to nonsmokers. All the analyses incorporated the weights derived from the complex sampling design. We used Stata v.13 to perform all analyses.

## RESULTS

### Opinions about smoking in different settings

**Figure 1:**
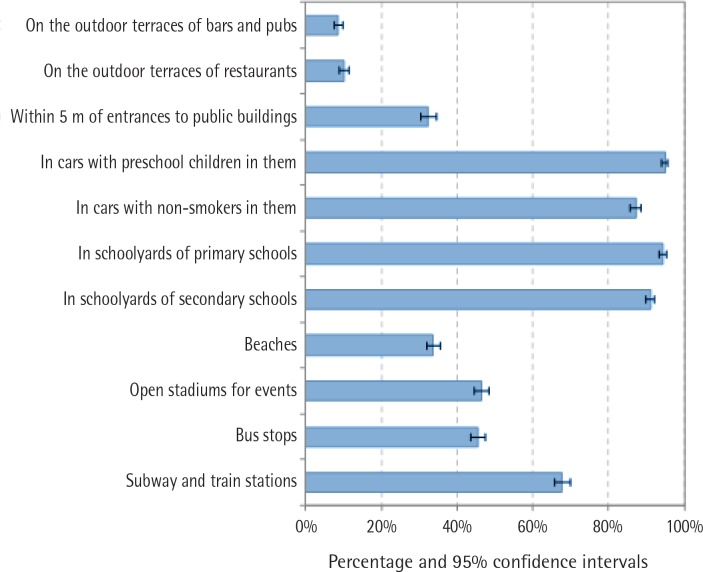
Support for smoke-free policies in different settings, 2016

In general, participants supported not allowing smoking indoors to a greater extent than in outdoor premises. More than 90% of smokers supported not allowing smoking in cars with preschool children and in schoolyards of primary and secondary schools, whereas lower support was found for not allowing smoking on outdoor terraces of bars and pubs (8.6%; 95% CI: 7.5%-9.8%) and on outdoor terraces of restaurants (10.1%; 95% CI: 8.9%-11.4%; Figure 1). While there was low variability by country in the support for not allowing smoking in places where there are minors (support >85% in all countries), greater variability was seen for open stadiums, in which the support varied from 24.4% in Greece to 78.4% in Poland, and for entrances of public buildings, where the support ranged from 20.5% in Spain to 69.4% in Hungary (Table 1).

**Table 1:** Support for smoke-free policies in different settings by country, 2016

	*Germany*		*Greece*		*Hungary*		*Poland*		*Romania*		*Spain*	

	*%*	*95% CI*	*%*	*95% CI*	*%*	*95% CI*	*%*	*95% CI*	*%*	*95% CI*	*%*	*95% CI*
On the outdoor terraces of bars and pubs	3.5	(1.6-5.3)	4.1	(2.2-6.0)	22.1	(16.8-27.3)	15.5	(12.1-18.8)	3.5	(2.0-5.0)	3.4	(2.1-4.7)
On the outdoor terraces of restaurants	8.5	(6.2-10.8)	5.2	(3.1-7.3)	24.3	(18.6-30.0)	15.3	(12.3-18.4)	3.2	(1.7-4.7)	4.2	(2.1-6.2)
Within 5 m of entrances to public buildings	21.4	(16.7-26.1)	26.8	(20.9-32.7)	69.4	(64.0-74.8)	26.2	(22.3-30.0)	30.4	(25.6-35.3)	20.5	(15.7-25.4)
In cars with preschool children in them	97.1	(95.6-98.6)	98.5	(97.7-99.4)	92.7	(89.7-95.6)	90.9	(88.3-93.4)	97.3	(96.2-98.4)	92.5	(89.8-95.1)
In cars with non-smokers in them	88.0	(84.7-91.2)	88.6	(85.5-91.6)	87.3	(83.5-91.1)	86.5	(82.6-90.4)	95.1	(93.2-97.0)	77.5	(73.4-81.5)
In schoolyards of primary schools	97.0	(95.7-98.2)	94.5	(91.6-97.5)	93.4	(90.6-96.2)	90.2	(87.5-92.9)	95.3	(93.0-97.7)	95.6	(93.1-98.1)
In schoolyards of secondary schools	88.1	(85.4-90.9)	92.9	(89.6-96.2)	92.7	(90.1-95.2)	85.7	(82.4-89.0)	95.2	(92.8-97.6)	91.2	(87.5-94.8)
Beaches	27.4	(22.6-32.1)	11.1	(8.1-14.1)	50.6	(44.0-57.2)	61.0	(56.2-65.9)	38.1	(33.0-43.1)	15.7	(12.3-19.2)
Open stadiums for events	32.5	(28.2-36.9)	24.4	(18.4-30.3)	65.9	(60.6-71.2)	78.4	(74.6-82.2)	47.0	(40.9-53.1)	31.3	(27.0-35.6)
Bus stops	26.8	(22.5-31.2)	25.8	(20.4-31.2)	64.9	(59.0-70.8)	72.1	(67.7-76.6)	50.3	(45.4-55.2)	33.0	(28.8-37.2)
Subway and train stations	53.6	(48.9-58.3)	67.5	(60.6-74.5)	69.7	(63.6-75.8)	79.5	(75.9-83.2)	73.1	(68.4-77.8)	63.2	(57.7-68.8)

### Support for a complete smoking ban inside public places

Overall, support for complete smoking bans was higher in hospitals and healthcare facilities (85.3%; 95% CI: 83.9%-86.8%) and in restaurants (68.0%; 95% CI: 66.0-69.9%; Table 2). Less support was declared for entertainment establishments (53.2%; 95% CI: 51.1%-55.4%) and drinking establishments (51.4%; 95% CI: 49.3%-53.5%). Of the six countries, smokers from Poland showed the highest support for a complete smoking ban. Overall, women showed greater support than men, as did respondents over 25 years of age. Respondents who had low nicotine dependence, as well as those who had attempted to quit in the last 12 months also showed greater support for a complete ban. Results by country are provided in the Supplementary Table S1.

**Table 2:** Support for a complete smoking ban inside public places by country, sociodemographic and smoking characteristics, 2016

	*Hospitals and healthcare facilities*			*Restaurants*			*Drinking establishments (pubs, bars)*			*Entertainment establishments (nightclub, discos)*		

	*n*	*%*	*95% CI*	*n*	*%*	*95% CI*	*n*	*%*	*95% CI*	*n*	*%*	*95% CI*
**All**	5081	85.3	83.9-86.8	3995	68.0	66.0-69.9	3021	51.4	49.3-53.5	3078	53.2	51.1-55.4
**Country**												
Germany	787	79.1	75.3-82.9	683	69.4	65.0-73.9	317	31.7	27.4-36.1	350	37.3	32.1-42.5
Greece	908	90.7	88.2-93.2	490	49.0	43.2-54.9	301	30.8	26.1-35.5	288	29.9	25.1-34.8
Hungary	807	81.4	76.7-86.1	702	72.0	67.1-76.9	507	51.1	44.7-57.5	559	57.0	50.8-63.2
Poland	881	91.2	89.1-93.4	775	81.2	77.5-84.8	674	72.0	67.5-76.6	677	72.0	67.4-76.5
Romania	799	79.5	75.2-83.8	601	61.1	56.7-65.5	545	54.5	49.8-59.1	548	55.5	50.6-60.3
Spain	899	90.0	87.1-92.8	744	75.3	70.7-79.8	677	68.9	64.2-73.6	656	67.5	63.1-71.9
**Sex**												
Men	2686	84.8	82.9-86.6	2103	67.9	65.6-70.2	1582	50.8	48.3-53.2	1597	52.3	49.7-54.9
Women	2395	86.1	84.4-87.7	1892	68.0	65.7-70.3	1439	52.2	49.8-54.7	1481	54.5	52.0-57.0
**Age (years)**												
18–24	426	83.5	79.7-87.4	325	61.3	56.3-66.3	226	43.0	37.7-48.2	212	40.8	35.7-45.8
25–39	1502	85.5	83.3-87.6	1216	69.6	66.7-72.6	923	53.5	50.3-56.7	937	54.8	51.6-58.0
40-54	1717	86.5	84.7-88.4	1330	69.1	66.7-71.6	966	51.2	48.4-54.1	1006	53.6	50.8-56.4
≥55	1436	84.2	81.8-86.5	1124	66.8	63.9-69.8	906	52.5	49.4-55.5	923	56.1	52.8-59.3
**Educational level**												
Low	1843	83.9	81.7-86.2	1469	67.8	64.9-70.7	1088	50.4	47.2-53.6	1131	53.0	49.6-56.3
Moderate	2641	85.7	84.1-87.4	2075	68.5	66.4-70.6	1595	53.0	50.5-55.5	1604	54.2	51.5-56.8
High	568	88.3	85.9-90.8	431	66.2	61.3-71.0	322	47.8	43.4-52.3	326	50.4	45.8-55.0
**Nicotine dependence**												
Low	2020	86.0	84.0-87.9	1723	74.6	72.2-76.9	1296	56.0	53.3-58.7	1327	58.7	55.9-61.4
Moderate	2351	84.6	82.7-86.4	1781	64.9	62.4-67.3	1364	49.8	47.1-52.5	1383	51.0	48.4-53.6
High	433	83.9	80.5-87.4	271	53.6	48.1-59.1	200	39.2	33.6-44.7	202	40.6	35.4-45.8
**Quit attempts in the last 12 months**												
Yes	927	87.1	84.5-89.8	781	73.9	70.6-77.2	613	57.8	54.3-61.3	625	60.2	56.7-63.8
No	4150	85.0	83.5-86.5	3210	66.7	64.6-68.8	2404	50.0	47.8-52.3	2449	51.8	49.5-54.0

### Beliefs about the harms of secondhand smoke to non-smokers

Overall, most smokers agreed that secondhand smoke is dangerous to non-smokers (77.6%; 95% CI: 75.8%-79.5%). This agreement was over 80% in most countries, except in Germany and Hungary (Table 3). The highest agreement was observed in Romania (88.3%; 95% CI: 85.1%-91.5%) and Spain (86.6%; 95% CI: 83.6%-89.6%), among women (79.6%; 95% CI: 77.6%-81.7%), in the 25-54 age groups (78.3%; 95% CI: 75.8%-80.8%), among respondents with high educational level (80.4%; 95% CI: 76.9%-84.0%), and among respondents with low nicotine dependence (78.1%; 95% CI: 75.7%-80.5%). The agreement with the statement about the harms of secondhand smoke to non-smokers was also higher among smokers who tried to quit in the last 12 months in all six countries (85.3%; 95% CI: 83.0%-87.7%; Table 3), although this difference was statistically significant only in Hungary (77.5%; 95% CI: 69.0%-86.0% vs 61.4%; 95% CI: 55.7%-67.1% of those without any attempt to quit smoking; Supplementary Table S2).

**Table 3:** Belief about the harms of secondhand smoke to non-smokers by country, sociodemographic and smoking characteristics, 2016

	*‘Cigarette smoke is dangerous to non-smokers’*								

	*Agree*			*Neither agree nor disagree*			*Disagree*		
	*n*	*%*	*95% CI*	*n*	*%*	*95% CI*	*n*	*%*	*95% CI*
**All**	4623	77.6	(75.8-79.5)	1003	17.1	(15.5-18.6)	341	5.3	(4.6-6.1)
**Country**									
Germany	663	64.7	(59.3-70.0)	241	26.0	(21.2-30.8)	92	9.3	(6.9-11.8)
Greece	816	80.3	(75.8-84.9)	130	15.0	(11.3-18.7)	52	4.7	(3.0-6.3)
Hungary	610	63.1	(57.7-68.5)	305	30.1	(25.0-35.2)	70	6.8	(4.7-9.0)
Poland	817	82.5	(78.8-86.1)	134	13.5	(10.5-16.6)	46	4.0	(2.5-5.5)
Romania	860	88.3	(85.1-91.5)	94	7.9	(5.7-10.2)	40	3.8	(2.2-5.4)
Spain	857	86.6	(83.6-89.6)	99	9.9	(7.3-12.5)	41	3.5	(2.4-4.6)
**Sex**												
Men	2397	76.1	(74.0-78.2)	559	18.3	(16.5-20.2)	191	5.6	(4.6-6.5)
Women	2226	79.6	(77.6-81.7)	444	15.4	(13.6-17.0)	150	5.0	(4.2-5.9)
**Age (years)**									
18–24	378	76.9	(73.1-80.6)	78	15.0	(11.6-18.5)	49	8.1	(5.7-10.5)
25–39	1365	78.3	(75.8-80.7)	299	16.8	(14.7-19.0)	100	4.9	(3.9-5.9)
40-54	1555	78.3	(75.8-80.8)	344	16.9	(14.7-19.1)	93	4.8	(3.7-5.9)
≥55	1325	76.1	(72.9-79.2)	282	18.3	(15.4-21.3)	99	5.6	(4.4-6.7)
**Educational level**									
Low	1639	74.6	(71.7-77.5)	399	19.2	(16.5-21.8)	148	6.2	(5.1-7.3)
Moderate	2438	79.2	(77.1-81.3)	489	15.8	(14.1-17.6)	164	5.0	(3.9-6.0)
High	517	80.4	(76.9-84.0)	109	15.4	(12.3-18.5)	27	4.2	(2.9-5.5)
**Nicotine dependence**									
Low	1843	78.1	(75.7-80.5)	385	16.9	(14.8-19.0)	122	5.0	(3.9-6.1)
Moderate	2145	77.1	(74.8-79.4)	486	17.5	(15.4-19.7)	164	5.4	(4.4-6.3)
High	376	75.6	(71.5-79.6)	93	17.7	(14.1-21.3)	38	6.7	(4.7-8.7)
**Quit attempts in the last 12 months**									
Yes	904	85.3	(83.0-87.7)	112	10.1	(8.1-12.1)	56	4.6	(3.4-5.8)
No	3714	76.0	(74.0-78.0)	891	18.5	(16.8-20.3)	285	5.5	(4.7-6.3)

We analysed the relationship between the respondents’ opinions about the places where smoking should be banned and the degree to which they believed that secondhand smoke was harmful to non-smokers. We observed that the support for a complete smoking ban was consistently higher among those who agreed with the statement that secondhand smoke is dangerous to non-smokers compared to those who did not agree with the statement; this pattern held true for all settings except for outdoor terraces of bars, pubs and restaurants, where no clear trend was observed (Table 4). The support for a complete smoking ban was lower than 35% for all outdoor settings, including outdoor terraces of bars and pubs, restaurants, within 5 m of entrances to public buildings, and at beaches (Table 4). In contrast, in all settings involving the presence of minors (in cars with preschool children and in schoolyards of primary and secondary schools), the support for complete smoking bans was higher than 80%, regardless whether the settings were indoors or outdoors (Table 4).

**Table 4:** Opinion about the danger of secondhand smoke to non-smokers according to the support for a smoking ban in different places, 2016

*Places where smoking should be banned*	*‘Cigarette smoke is dangerous to non-smokers’*											

	*All*			*Agree*			*Neither agree nor disagree*			*Disagree*		
	*n*	*%*	*95% CI*	*n*	*%*	*95% CI*	*n*	*%*	*95% CI*	*n*	*%*	*95% CI*
**Opinion about the places where smoking should be banned**												
On the outdoor terrace of bars and pubs	531	8.6	(7.5-9.8)	391	8.0	(6.9-9.1)	103	10.4	(8.0-12.9)	26	9.7	(5.6-13.8)
On the outdoor terrace of restaurants	617	10.1	(8.9-11.4)	452	9.4	(8.1-10.6)	122	12.3	(9.6-15.1)	29	10.0	(6.5-13.5)
Within 5 m of the entrance to public buildings	1897	32.4	(30.3-34.5)	1479	32.7	(30.3-35.1)	310	32.1	(28.2-35.9)	92	27.9	(22.3-33.5)
In cars with preschool children in them	5601	94.8	(94.0-95.7)	4362	95.4	(94.5-96.3)	914	94.6	(92.9-96.2)	287	87.2	(83.1-91.2)
In cars with non-smokers in them	5094	87.2	(85.8-88.6)	4035	89.0	(87.5-90.4)	782	82.7	(79.8-85.5)	239	73.4	(67.6-79.2)
In schoolyards of primary schools	5628	94.4	(93.3-95.4)	4375	94.9	(93.7-96.0)	914	93.4	(91.5-95.3)	298	89.9	(86.0-93.8)
In schoolyards of secondary schools	5436	91.0	(89.8-92.2)	4249	91.9	(90.6-93.2)	878	89.8	(87.5-92.1)	271	80.6	(75.3-85.9)
Beaches	1992	33.7	(31.9-35.6)	1574	34.2	(32.2-36.2)	313	33.3	(29.3-37.2)	86	25.5	(19.9-31.0)
Open stadiums for events such as football, etc.	2740	46.5	(44.4-48.5)	2156	47.6	(45.3-49.8)	434	44.1	(40.0-48.2)	125	35.6	(29.9-41.3)
Bus stops	2695	45.4	(43.5-47.4)	2167	47.2	(45.1-49.3)	388	40.4	(36.1-44.7)	120	35.3	(29.0-41.6)
Subway and train stations	3990	67.8	(65.7-70.0)	3193	70.1	(67.8-72.4)	587	60.9	(56.4-65.4)	182	56.4	(49.7-63.1)
**Opinion about a complete smoking ban inside:**												
Restaurants	3995	68.0	(66.0-69.9)	3187	70.3	(68.3-72.2)	596	61.1	(56.5-65.8)	191	57.3	(50.7-63.8)
Drinking establishments as pubs and bars	3021	51.4	(49.3-53.5)	2480	54.7	(52.4-56.9)	401	40.5	(36.0-45.1)	123	38.7	(32.2-45.3)
Entertainment establishments such as nightclubs and discos	3078	53.2	(51.1-55.4)	2508	56.1	(53.9-58.4)	424	44.0	(39.3-48.7)	126	39.7	(32.9-46.5)
Hospitals and healthcare facilities	5081	85.3	(83.9-86.8)	3984	86.9	(85.5-88.3)	809	81.7	(78.1-85.3)	255	72.8	(67.3-78.4)

## DISCUSSION

This study examined smokers’ opinions about smoking bans in a number of outdoor and indoor settings, some of which are not included in national smoke-free legislation and where non-smokers could be potentially exposed to secondhand smoke. Most smokers from Germany, Greece, Hungary, Poland, Romania, and Spain agreed with regulating smoking in enclosed or semi-enclosed settings, in the presence of non-smokers and particularly in the presence of minors. Similarly, smokers in the six EU MS declared high support for smoking bans in hospitals and healthcare settings. This finding is in line with some previous studies^17-19^ and could be potentially explained by the increased perception of harmful effects of passive exposure in these settings, as well as a wider spread of smoke-free regulations in healthcare settings already in place^20^.

We found higher support for banning smoking in restaurants than in drinking or entertainment establishments. Previous studies based on other ITC surveys in Europe have also found this trend; the support for smoking regulations was about 40:80% in restaurants and about 10-45% in bars after the implementation of smoke-free regulations in France, Ireland, the United Kingdom, Scotland, Germany and the Netherlands^4,6,8,9^. In our survey, when asked about open areas in these settings, we found less support for banning smoking in outdoor terraces of restaurants, bars and pubs compared to all the other outdoor settings assessed (entrances to public buildings, beaches, bus stops, open stadiums, and schoolyards). In another ITC survey conducted in France, it was observed that the indoor smoking legislation implemented in 2008 moved smokers to outdoor settings; nevertheless, the ban was also associated with increased non-smoking behaviour and higher support for partial (64.6%) rather than total bans (10.2%)^21^. Another different study conducted in Barcelona, Spain, also showed that the smoking legislation indoors moved smokers to outdoor settings (terraces of pubs and bars), with non-smokers reporting exposure to secondhand smoke in most outdoor settings in which smokers reported smoking after a national comprehensive ban; also, there was great support for smoke-free areas outdoors, particularly for areas in which children are present, for grounds of healthcare centres and, to a lesser extent, for outdoor areas of public transportation, sport centres and university campuses^22^.

In our data, higher support for smoking bans in enclosed public places was observed among smokers of older age, lower nicotine dependence, and having previous quit attempts. Some studies have identified the same smoking-related variables, among others, being associated with the compliance with smoke-free legislation^23,24^; this suggests that smokers with these characteristics are more likely to support and comply with smoke-free legislation in enclosed public settings. Also, our data indicated an inverse trend in the support for smoking bans in all indoor settings, according to the smokers’ belief about the harms of secondhand smoke to non-smokers, with the highest support among those who agreed with this statement. This result is consistent with findings from the ITC Europe Surveys conducted pre- and post-legislation in France, Germany, Ireland, and the Netherlands, showing that smokers more supportive of smoking bans were more aware of the harmful effects of secondhand smoke exposure^25^. It was also found that high support for smoking bans and a strong belief about the harmful effects of smoking to others was negatively associated with smoking in bars after the legislation^25^.

Overall, smokers’ support for smoking regulations may reflect the current state of the smoke-free legislation^9,20^. In other words, smokers report higher support for bans in settings that are already totally or partially regulated by local or national laws, or are located close to places where smoking is currently regulated (e.g. schoolyards or cars with children); and *vice versa*, the less rigorous the smoking regulation (e.g. in terraces of bars and restaurants), the lower the support for smoking bans. This might suggest a trend of the current regulation to shape smokers’ beliefs and support for smoking bans, which is an important reflection of a feasible implementation of smoke-free legislation in sensitive settings not affected by such regulation. The enforcement of smoke-free regulations in indoor and outdoor settings where minors are present could be regarded as a priority action, not only for protecting the health of children and adolescents from the exposure to secondhand smoke but also because smokers’ support and adherence to such measures is more likely. Following the WHO FCTC recommendations, policymakers should promote the adoption and implementation of effective legislative, executive, administrative or other measures, providing protection from exposure to secondhand smoke in diverse public places and, if appropriate, private settings such as cars with minors.

### Limitations and strengths

The main limitation of this study is the use of self-reported information, particularly when addressing smokers, with a potential information bias due to social desirability. Also, the cross-sectional nature of the study precludes any causal relationship among the studied variables. Further longitudinal analyses will, however, allow for the identification of changes and trends over time. On the other hand, the main strength of this study is the use of nationally representative samples of smokers in six EU MS, five of them with novel data regarding smokers’ attitudes towards smoke-free regulations using comprehensive and well-established ITC survey methods.

## CONCLUSIONS

Our data suggest that smokers support smoke-free policies mostly in indoor settings as well as in settings where minors are present. There is less support for smoking bans in outdoor settings, particularly in outdoor terraces of leisure facilities. These results are consistent with their beliefs about the harms of secondhand smoke to non-smokers. Lower support for smoke-free regulations in outdoor settings indicates that further efforts are required to increase smokers’ awareness about the potential exposure to secondhand smoke in specific outdoor areas such as terraces as well as near entrances of public buildings.
