# Supplementary material for: Correlates of the support for smoke-free policies among smokers: A cross-sectional study in six European countries of the EUREST-PLUS ITC EUROPE SURVEYS
Source: Tob Induc Dis. 2019 Mar 27;16:A17. doi: 10.18332/tid/103918 (PMC6661849; doi:10.18332/tid/103918)
Supplement: Supplementary file 1 [file TID-16-A17-s1.pdf]

Supplementary Table S1. Support for a complete smoking ban inside public places according to sociodemographic and smoking characteristics by country, 2016.

GERMANY

	Hospitals and health care facilities			Restaurants			Drinking establishments (pubs, bars)			Entertainment establishments (nightclub, discos)		
	n	%	95% CI	n	%	95% CI	n	%	95% CI	n	%	95% CI
**Sex**												
Men	391	78.0	73.3–82.6	343	70.2	65.0–75.4	160	31.1	26.2–36.0	169	34.9	29.1–40.7
Women	396	80.9	77.0–84.9	340	68.2	63.1–73.4	157	32.8	27.7–37.8	181	41.2	35.7–46.7
**Age (years)**												
18-24	65	74.2	63.7–84.7	60	68.4	58.2–78.7	28	34.7	23.4–46.0	30	36.3	26.6–45.9
25-39	214	78.2	72.7–83.8	178	63.3	56.2–70.4	75	26.0	20.0–32.0	88	31.8	25.2–38.4
40-54	280	81.7	76.8–86.7	250	74.9	68.8–80.9	106	32.3	26.6–37.9	119	37.6	31.6–43.6
≥55	228	77.9	71.3–84.5	195	68.2	61.5–74.9	108	35.3	28.9–41.8	113	42.7	34.3–51.1
**Educational level**												
Low	390	78.1	73.0–83.2	349	69.3	63.9–74.6	173	33.5	27.1–40.0	192	38.9	32.4–45.4
Moderate	335	80.3	75.6–84.9	284	70.4	64.8–76.1	122	30.5	24.9–36.1	134	35.8	29.3–42.2
High	61	79.9	71.3–88.5	48	64.2	50.0–78.4	21	26.4	15.8–37.0	23	35.0	22.4–47.6
**Nicotine dependence**												
Low	340	76.7	70.9–82.5	318	74.0	68.5–79.6	139	32.2	27.1–37.2	161	39.9	34.1–45.8
Moderate	298	80.5	76.1–84.9	253	67.9	62.1–73.7	124	32.8	26.0–39.5	130	35.3	28.4–42.3
High	41	63.6	48.6–78.7	31	48.7	34.8–62.5	9	14.0	5.2–22.8	12	18.4	8.9–27.8
**Quit attempts in the last 12 months**												
Yes	148	82.4	76.7–88.2	139	77.1	70.6–83.6	75	39.8	31.8–47.7	88	51.0	43.7–58.4
No	639	78.4	74.4–82.5	544	67.8	63.0–72.7	242	30.0	25.5–34.6	262	34.4	29.0–39.8

GREECE

	Hospitals and health care facilities			Restaurants			Drinking establishments (pubs, bars)			Entertainment establishments (nightclub, discos)		
	**n**	**%**	**95% CI**	**n**	**%**	**95% CI**	**n**	**%**	**95% CI**	**n**	**%**	**95% CI**
**Sex**												
Men	500	91.0	87.6–94.5	272	49.3	42.6–56.0	160	30.0	24.6–35.4	153	28.9	23.4–34.4
Women	408	90.2	86.7–93.8	218	48.7	41.8–55.7	141	31.8	25.9–37.7	135	31.0	25.0–37.1
**Age (years)**												
18-24	54	90.4	82.7–98.1	31	47.4	34.7–60.2	15	21.1	10.4–31.7	13	19.3	9.1–29.6
25-39	230	89.1	84.7–93.5	132	52.7	44.1–61.2	80	32.7	24.9–40.5	77	31.8	24.0–39.5
40-54	356	93.5	91.0–96.0	191	50.6	43.8–57.3	113	31.7	25.2–38.1	111	31.3	24.8–37.9
≥55	268	88.7	84.9–92.6	136	43.6	35.9–51.4	93	30.8	24.7–36.8	87	29.3	23.0–35.7
**Educational level**												
Low	274	89.1	84.3–93.9	138	43.8	34.2–53.5	92	29.4	21.5–37.4	87	27.0	18.8–35.2
Moderate	446	91.2	88.6–93.9	241	49.4	42.9–56.0	136	29.8	24.0–35.5	135	30.0	24.1–35.9
High	186	91.9	88.6–95.2	111	55.7	46.3–65.1	73	35.6	28.2–42.9	66	34.2	26.7–41.6
**Nicotine dependence**												
Low	289	89.6	86.0–93.3	180	56.3	48.7–63.9	106	34.8	28.4–41.3	105	34.8	28.1–41.5
Moderate	436	90.9	87.4–94.4	216	45.0	38.7–51.4	135	29.3	23.3–35.3	129	28.3	22.2–34.4
High	156	91.8	88.0–95.7	69	39.7	29.9–49.5	47	25.9	17.6–34.1	43	24.4	15.7–33.0
**Quit attempts in the last 12 months**												
Yes	147	93.6	89.3–97.8	104	64.8	55.3–74.4	59	36.1	28.5–43.7	53	34.1	26.1–42.2
No	761	90.2	87.3–93.0	386	46.2	40.1–52.4	242	29.9	24.8–35.1	235	29.2	24.0–34.4

HUNGARY

	Hospitals and health care facilities			Restaurants			Drinking establishments pubs, bars			Entertainment establishments nightclub, discos		
	**n**	**%**	**95% CI**	**n**	**%**	**95% CI**	**n**	**%**	**95% CI**	**n**	**%**	**95% CI**
**Sex**												
Men	419	80.0	74.6–85.4	362	70.7	65.2–76.2	260	50.1	42.6–57.6	291	57.1	50.3–63.9
Women	388	83.4	78.5–88.4	340	73.9	68.3–79.4	247	52.6	46.1–59.0	268	56.7	49.9–63.6
**Age (years)**												
18-24	50	80.7	67.4–94.1	46	72.5	58.6–86.4	34	52.4	35.7–69.0	38	58.7	42.5–75.0
25-39	226	80.7	73.4–88.1	211	75.6	68.3–82.9	152	54.5	46.6–62.4	168	60.0	52.1–67.9
40-54	287	83.0	77.8–88.2	243	71.4	65.7–77.0	167	48.3	40.0–56.7	186	53.7	45.5–62.0
≥55	244	80.4	73.7–87.1	202	67.3	60.4–74.2	154	49.6	42.5–56.8	167	56.4	49.2–63.5
**Educational level**												
Low	512	83.1	77.8–88.3	443	72.6	67.0–78.2	333	53.5	46.5–60.5	368	59.4	52.2–66.7
Moderate	242	78.7	71.2–86.1	212	70.7	63.7–77.7	141	46.5	38.2–54.9	155	52.6	44.5–60.6
High	52	78.1	66.3–89.8	47	73.8	60.8–86.7	32	48.0	34.3–61.8	35	52.4	38.6–66.2
**Nicotine dependence**												
Low	290	84.8	79.7–89.9	258	75.6	69.9–81.3	184	54.3	46.0–62.7	196	58.4	50.1–66.7
Moderate	454	79.1	73.2–85.1	393	70.0	64.1–75.9	288	50.4	43.3–57.6	322	56.7	50.1–63.3
High	53	81.2	71.4–91.1	42	67.6	54.7–80.6	27	38.2	23.5–53.0	32	48.6	34.9–62.3
**Quit attempts in the last 12 months**												
Yes	91	84.7	77.8–91.6	84	76.3	68.1–84.5	67	61.9	51.7–72.2	75	69.6	60.4–78.8
No	716	81.0	76.1–85.9	618	71.5	66.2–76.7	440	49.8	43.4–56.3	484	55.5	49.0–61.9

POLAND

	Hospitals and health care facilities			Restaurants			Drinking establishments pubs, bars			Entertainment establishments nightclub, discos		
	n	%	95% CI	n	%	95% CI	n	%	95% CI	n	%	95% CI
**Sex**												
Men	412	90.5	87.3–93.6	353	79.0	74.3–83.7	293	68.8	63.3–74.3	292	68.1	62.2–73.9
Women	469	92.2	90.0–94.5	422	83.9	80.3–87.4	381	76.0	71.2–80.7	385	76.6	71.8–81.5
**Age (years)**												
18-24	58	81.1	70.5–91.7	49	67.4	54.3–80.4	40	60.6	47.9–73.4	36	50.6	38.8–62.5
25-39	305	92.2	89.2–95.2	269	81.3	76.2–86.5	232	71.7	64.8–78.5	229	71.2	64.5–77.9
40-54	249	92.2	88.7–95.6	222	82.4	77.4–87.5	192	73.0	66.8–79.1	198	73.4	67.3–79.5
≥55	269	91.9	88.3–95.5	235	83.6	78.9–88.2	210	74.7	68.1–81.2	214	77.4	71.8–83.1
**Educational level**												
Low	110	89.8	83.8–95.7	85	71.0	62.4–79.6	75	64.2	54.1–74.4	76	64.0	53.9–74.1
Moderate	660	91.8	89.9–93.7	586	82.4	78.5–86.3	516	74.6	69.9–79.3	510	73.4	68.3–78.4
High	95	87.6	81.6–93.6	93	84.8	79.5–90.0	75	66.2	56.8–75.5	82	73.9	66.3–81.4
**Nicotine dependence**												
Low	325	91.0	87.2–94.7	306	87.9	83.9–92.0	269	78.6	72.8–84.4	272	80.3	74.9–85.7
Moderate	443	91.4	88.8–94.0	384	79.8	75.3–84.4	329	70.1	64.7–75.6	328	69.4	63.9–74.9
High	54	93.9	86.4–100	37	61.4	46.7–76.2	29	50.0	35.9–64.1	30	52.1	37.1–67.2
**Quit attempts in the last 12 months**												
Yes	153	88.9	82.7–95.1	137	80.3	72.8–87.7	121	70.6	62.0–79.3	124	72.2	63.4–81.0
No	724	91.8	89.7–93.9	634	81.4	77.6–85.2	549	72.3	67.6–77.0	549	71.9	67.3–76.5

ROMANIA

	Hospitals and health care facilities			Restaurants			Drinking establishments pubs, bars			Entertainment establishments nightclub, discos		
	**n**	**%**	**95% CI**	**n**	**%**	**95% CI**	**n**	**%**	**95% CI**	**n**	**%**	**95% CI**
**Sex**												
Men	470	80.6	75.4–85.8	367	63.3	57.6–69.0	327	56.0	50.4–61.6	326	56.6	50.5–62.7
Women	329	78.0	72.5–83.5	234	57.9	51.5–64.2	218	52.3	46.3–58.3	222	53.9	47.7–60.1
**Age (years)**												
18-24	94	82.2	74.7–89.6	57	49.7	38.7–60.8	48	38.1	27.0–49.1	43	34.5	24.0–44.9
25-39	243	81.7	76.4–87.0	191	67.6	60.8–74.4	172	60.3	53.0–67.6	169	60.6	52.7–68.4
40-54	255	78.0	72.4–83.5	177	55.1	49.5–60.8	161	50.5	44.9–56.1	170	53.4	47.4–59.4
≥55	207	75.0	67.0–83.0	176	67.2	58.5–75.8	164	62.6	53.4–71.9	166	66.1	56.8–75.3
**Educational level**												
Low	195	82.2	74.8–89.7	150	65.1	57.4–72.8	133	58.2	50.2–66.3	135	59.3	50.5–68.2
Moderate	498	76.7	71.7–81.7	378	60.0	55.6–64.5	347	53.9	49.1–58.6	347	54.8	49.4–60.2
High	98	89.4	84.8–94.0	67	59.6	47.2–72.0	60	51.7	40.9–62.5	61	53.3	43.8–62.8
**Nicotine dependence**												
Low	312	82.5	77.1–88.0	254	69.4	64.0–74.7	225	61.1	55.1–67.1	230	62.9	56.8–69.1
Moderate	380	77.3	72.1–82.5	274	57.0	51.2–62.7	252	50.3	44.6–55.9	249	50.9	44.3–57.5
High	63	79.0	70.2–87.8	38	49.2	34.9–63.4	37	48.9	33.6–64.3	36	47.6	33.8–61.4
**Quit attempts in the last 12 months**												
Yes	233	84.6	78.3–90.8	186	69.6	63.2–76.1	170	62.5	56.0–69.0	167	62.0	55.4–68.6
No	566	77.7	73.0–82.5	415	58.0	52.7–63.4	375	51.5	46.2–56.8	381	53.1	47.6–58.5

SPAIN

	Hospitals and health care facilities			Restaurants			Drinking establishments pubs, bars			Entertainment establishments nightclub, discos		
	**n**	**%**	**95% CI**	**n**	**%**	**95% CI**	**n**	**%**	**95% CI**	**n**	**%**	**95% CI**
**Sex**												
Men	494	89.8	86.1–93.5	406	74.1	68.9–79.3	382	69.5	64.2–74.8	366	67.6	62.6–72.6
Women	405	90.3	87.4–93.1	338	76.8	71.5–82.2	295	68.1	62.7–73.5	290	67.3	62.0–72.7
**Age (years)**												
18-24	105	90.1	84.2–96.0	82	66.9	55.9–77.8	61	51.3	40.8–61.8	52	45.9	35.7–56.2
25-39	284	90.8	87.7–93.9	235	74.5	68.1–81.0	212	67.6	60.6–74.6	206	65.2	57.9–72.5
40-54	290	90.3	85.7–94.9	247	80.1	74.1–86.1	227	74.5	68.0–80.9	222	73.8	67.6–79.9
≥55	220	88.0	83.3–92.8	180	72.3	67.3–77.3	177	70.9	65.4–76.4	176	71.9	65.6–78.1
**Educational level**												
Low	362	87.4	82.8–92.0	304	76.1	70.1–82.0	282	71.1	65.5–76.7	273	69.9	64.4–75.3
Moderate	460	91.7	89.0–94.4	374	74.6	69.6–79.6	333	66.5	60.5–72.5	323	65.0	59.3–70.7
High	76	94.4	90.2–98.7	65	75.3	64.2–86.4	61	71.6	59.9–83.3	59	69.5	57.7–81.3
**Nicotine dependence**												
Low	464	91.7	89.0–94.3	407	80.9	75.9–85.9	373	74.4	69.2–79.7	363	73.1	68.3–77.9
Moderate	340	89.0	84.6–93.5	261	69.4	62.8–75.9	236	63.0	56.3–69.7	225	60.9	54.1–67.8
High	66	84.1	74.4–93.8	54	71.0	59.9–82.0	51	67.1	55.6–78.5	49	66.0	54.7–77.4
**Quit attempts in the last 12 months**												
Yes	155	90.0	81.8–98.1	131	77.9	68.6–87.1	121	72.6	63.5–81.7	118	71.6	62.4–80.8
No	744	90.0	87.4–92.5	613	74.7	69.9–79.5	556	68.1	63.2–73.0	538	66.6	62.3–70.9

Supplementary Table S2. Belief about the harmfulness of secondhand smoke to non-smokers according to sociodemographic and smoking characteristics by country, 2016.

	Germany			Greece			Hungary			Poland			Romania			Spain		
	**n**	**%**	**95% CI**	**n**	**%**	**95% CI**	**n**	**%**	**95% CI**	**n**	**%**	**95% CI**	**n**	**%**	**95% CI**	**n**	**%**	**95% CI**
**Sex**																		
Men	324	63.1	56.9–69.3	437	78.8	73.2–84.3	306	61.8	56.0–67.7	379	81.2	76.9–85.6	498	87.9	84.7–91.1	453	84.7	80.9–88.5
Women	339	67.1	61.7–72.5	379	82.1	77.3–86.9	304	64.8	58.8–70.8	438	84.0	80.1–87.9	362	88.8	84.6–93.1	404	89.1	85.4–92.8
**Age (years)**																		
18-24	56	65.5	54.9–76.0	44	72.8	60.9–84.8	34	66.8	56.7–76.8	54	75.2	65.5–85.0	91	84.0	76.6–91.3	99	87.8	81.6–93.9
25-39	194	68.0	62.5–73.5	206	80.2	73.3–87.0	164	60.6	53.2–68.0	270	81.6	77.4–85.7	259	88.5	84.1–92.9	272	88.2	83.8–92.6
40-54	220	62.0	54.2–69.8	321	85.9	81.3–90.4	222	63.5	56.1–70.9	238	85.7	81.0–90.4	278	89.2	85.1–93.3	276	85.4	81.1–89.7
≥55	193	64.9	56.8–72.9	245	75.5	66.9–84.1	190	64.5	57.0–72.1	255	82.3	75.6–88.9	232	89.9	84.9–95.0	210	85.8	81.7–89.9
**Educational level**																		
Low	334	63.5	57.5–69.5	249	78.0	67.7–88.4	389	65.0	58.9–71.1	97	79.2	70.2–88.1	219	93.4	90.0–96.8	351	87.0	82.9–91.0
Intermediate	276	65.4	57.5–73.2	393	78.8	73.6–84.0	182	60.2	53.2–67.2	617	83.1	79.5–86.8	538	87.6	83.8–91.4	432	86.1	82.4–89.8
High	51	67.2	56.8–77.7	172	87.4	81.7–93.0	37	55.5	41.2–69.9	91	82.4	74.7–90.0	94	83.5	73.3–93.6	72	87.4	79.1–95.6
**Nicotine dependence**																		
Low	274	59.9	52.3–67.5	267	80.8	73.4–88.2	235	69.1	61.8–76.4	306	84.9	80.9–88.9	313	86.5	82.1–90.9	448	88.7	85.7–91.7
Moderate	266	68.3	62.2–74.4	385	78.9	73.6–84.2	329	59.2	52.4–66.0	418	82.0	77.6–86.4	432	90.2	86.7–93.8	315	84.1	79.1–89.2
High	31	52.7	40.1–65.4	135	80.3	72.8–87.9	40	66.4	53.2–79.7	38	70.5	57.6–83.4	68	84.1	75.5–92.7	64	84.7	76.6–92.8
**Quit attempts in the last 12 months**																		
Yes	135	74.0	66.7–81.4	138	86.7	81.0–92.4	80	77.5	69.0–86.0	154	87.1	81.9–92.3	246	92.4	88.5–96.3	151	87.3	81.6–93.0
No	528	62.7	57.0–68.5	678	79.2	74.3–84.1	530	61.4	55.7–67.1	659	81.5	77.5–85.5	613	86.8	83.1–90.5	706	86.4	83.4–89.5

© 2019 Fu M.
